# Linking consumer physiological status to food-web structure and prey food value in the Baltic Sea

**DOI:** 10.1007/s13280-019-01201-1

**Published:** 2019-06-05

**Authors:** Agnes M. L. Karlson, Elena Gorokhova, Anna Gårdmark, Zeynep Pekcan-Hekim, Michele Casini, Jan Albertsson, Brita Sundelin, Olle Karlsson, Lena Bergström

**Affiliations:** 1 Department of Ecology, Environment and Plant Science, Stockholm University, Svante Arrhenius väg 21 A, 106 91 Stockholm, Sweden; 2 Stockholm University Baltic Sea Centre, Stockholm University, Stockholm, Sweden; 3 Department of Environmental Science and Analytical Chemistry, Stockholm University, 106 91 Stockholm, Sweden; 4 Department of Aquatic Resources, Institute of Coastal Research, Swedish University of Agricultural Sciences, Skolgatan 6, 742 42 Öregrund, Sweden; 5 Department of Aquatic Resources, Institute of Marine Research, Swedish University of Agricultural Sciences, Turistgatan 5, 453 30 Lysekil, Sweden; 6 Umeå Marine Science Centre, Umeå University, Norrbyn 557, 905 71 Hörnefors, Sweden; 7 Department of Environmental Research and Monitoring, Swedish Museum of Natural History, P.O. 50007, 104 05 Stockholm, Sweden

**Keywords:** Benthic–pelagic coupling, Benthivore, Ecological indicator, Long-term time series, Piscivore, Zooplanktivore

## Abstract

Declining physiological status in marine top consumers has been observed worldwide. We investigate changes in the physiological status and population/community traits of six consumer species/groups in the Baltic Sea (1993–2014), spanning four trophic levels and using metrics currently operational or proposed as indicators of food-web status. We ask whether the physiological status of consumers can be explained by food-web structure and prey food value. This was tested using partial least square regressions with status metrics for gray seal, cod, herring, sprat and the benthic predatory isopod *Saduria* as response variables, and abundance and food value of their prey, abundance of competitors and predators as predictors. We find evidence that the physiological status of cod, herring and sprat is influenced by competition, predation, and prey availability; herring and sprat status also by prey size. Our study highlights the need for management approaches that account for species interactions across multiple trophic levels.

**Electronic supplementary material:**

The online version of this article (10.1007/s13280-019-01201-1) contains supplementary material, which is available to authorized users.

## Introduction

The physiological status of keystone species is an important characteristic of overall food-web status because it determines populations’ potential for growth and reproduction and, hence, their long-term sustainability (Kadin et al. 2012). It may also have direct economic consequences, such as for the value of commercial fisheries (Marshall et al. 2000). Physiological status can be measured in several ways, and different approaches may be preferential for different species, such as relative body condition (based on weight, size or fat content) or reproductive output. In recent decades, declining breeding success and body condition have been observed in marine top consumers worldwide, and have been attributed to various changes in the food-web (e.g. Trites and Donnelly 2003; Österblom et al. 2008; Bogstad et al. 2015; Harwood et al. 2015; Casini et al. 2016).

Several human-induced pressures and environmental changes have been related to impacts on the physiological status of commercial fish, via direct or indirect pathways. In the Baltic Sea, main anthropogenic pressures include overfishing, eutrophication, and climate change (Andersson et al. 2015; Elmgren et al. 2015). Fishing can directly influence the size structure of commercial target species (Östman et al. 2014), resulting in reduced body size and growth, or decreased size at maturation (Vainikka et al. 2009). Overfishing may also lead to cascading effects on lower trophic levels (e.g. Casini et al. 2008), which in the Baltic Sea has been seen to lead to enhanced competition for food among forage fish when these are released from predation, resulting in reduced physiological condition in sprat and herring (Casini et al. 2010). Hence, human-induced alterations of food-web structure can affect the physiological status of species.

Structural changes due to bottom-up processes may also affect the physiological status of species, including consumers. Whereas top-down effects primarily act via changes in the abundance of predators, bottom-up effects can be mediated through changes in both prey availability and quality as food. Experimentally modified elemental and biochemical composition of phytoplankton translates into lower food quality for zooplankton, and, ultimately, can lead to reduced growth of zooplanktivores, such as larval herring and trout (Malzahn et al. 2007; Taipale et al. 2018). In the field, however, the quantity and size of prey seem to be more decisive for juvenile clupeid fish than their fatty acid composition (Peters et al. 2015). So far, we are not aware of any studies evaluating the influence of prey quality at several trophic levels across an entire food-web. The Baltic Sea, with its uniquely low taxonomic diversity (Elmgren and Hill 1997), provides an opportunity to test the importance of food-web structure and food value of prey, respectively, on the physiological status of consumers using monitoring-based time series data covering multiple trophic levels.

Here, we study long-term changes in the physiological status of consumers from four trophic levels in the Baltic Sea, and test whether these can be attributed to top-down or bottom-up changes in food-web structure (as represented by abundance of predators, competitors and prey) and/or food value (physiological status, or energy content of prey). We gather metrics on the physiological status of gray seal (*Halichoerus grypus*), cod (*Gadus morhua*), herring (*Clupea harengus*), and sprat (*Sprattus sprattus*). Blubber in seals is a layer of lipid-rich tissue between the epidermis and the underlying muscles, which acts as a storage of metabolic energy, and is important not only for individual survival but also for reproduction (Harding et al. 2005; Helcom 2018). In fish, lipids is the main source of energy. In forage fish, such as sprat and herring, the lipid content is on average 34% of the body mass, and females with higher lipid content have higher egg survival (Laine and Rajasilta 1999). Previous studies have seen that lipid content and blubber thickness are influenced by prey quality (Røjbek et al. 2014; Kauhala et al. 2017; Rajasilta et al. 2019), while body size in fish also responds to size-selective predation (e.g. Vainikka et al. 2009).

The study focuses on the years 1993–2014, which corresponds to an ecologically relatively stable time period compared to the preceding years, which were characterized by strong shifts in species composition in the pelagic food-web (Casini et al. 2008). We predict that (i) high prey availability and (ii) high prey food value have a positive influence on the physiological status of consumers at higher trophic levels via bottom-up processes, that (iii) high abundances of intra- or interspecific competitors have negative effects on the physiological status of consumers due to increased competition for food, and that (iv) predation might have either positive or negative effects on the physiological status of prey, due to selective mortality (depending on whether larger, smaller or individuals in bad condition are eaten first), or positive effects by reducing intra-specific competition.

## Materials and Methods

### Study system

**Fig. 1:**
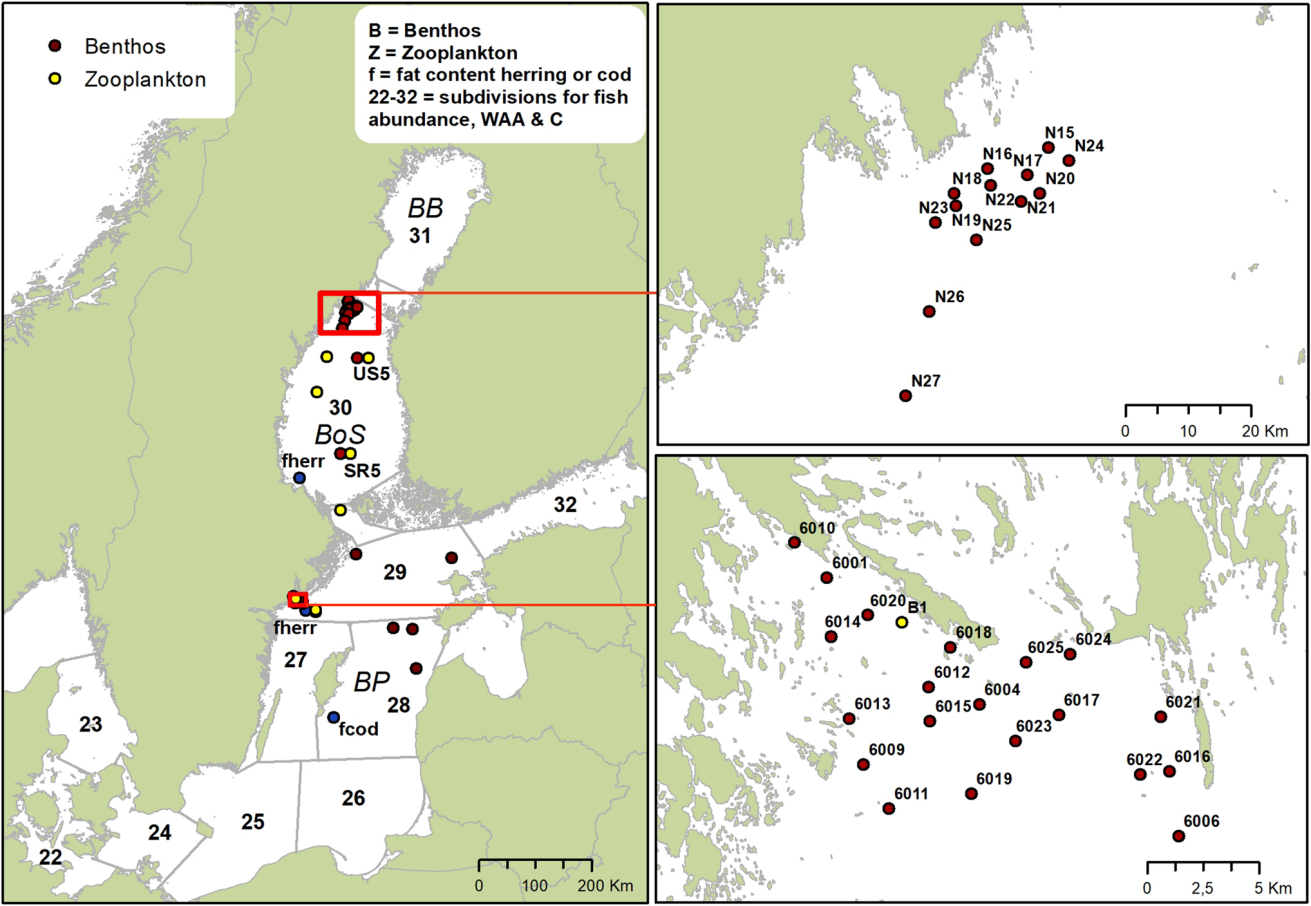
Map of the Baltic Sea with its major basins; Bothnian Bay (BB), Bothnian Sea (BoS) and the Baltic Proper (BP), showing the used sampling stations (see inserted legend). Fish data are assembled based on ICES subdivisions (SD), shown as numbers in the left panel; the cod stock is distributed over SD 25–29 (i.e. the Baltic Proper), the BP stock of herring occurs in SD 25–29 and 32, while the herring in the BoS is a separate stock (SD 30). Sprat and gray seal represent the same stock/population in all of the Baltic Sea (SD 22–32). Zoomed-in maps show zooplankton and benthos stations in the Askö area (lower right panel) and in the northern Bothnian Sea (upper right panel). Data on *M. affinis* embryo viability originate from stations 6004, 6019, 6020, 6022 and 6025 in BP, and from N19, N25, N26, N27 and US5 in BoS. The five benthos stations in the left panel (BP) are referred to as open sea stations. See text and Table 1 for details on monitoring programs and Table S1 for details and meta-data on sampling stations

The Baltic Sea is the world’s largest brackish water system, and is naturally species-poor due to its low salinity (Elmgren and Hill 1997). In this study, we analyzed changes in physiological status across four trophic levels in two sub-systems; the basins of the Baltic Proper (BP) and the Bothnian Sea (BoS) (Fig. 1). These systems differ in hydrological conditions, with an average surface salinity of 6–8 in BP and 4–6 in BoS, and a mean annual surface temperature of 9 °C in BP versus 7 °C in BoS.

**Fig. 2:**
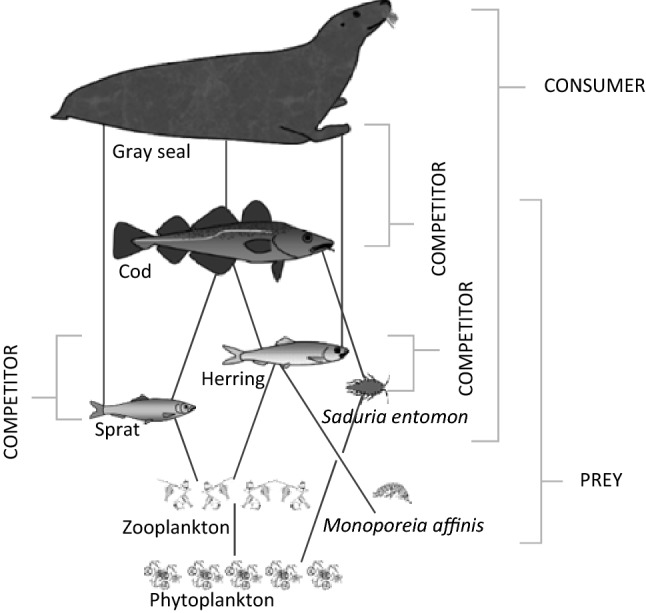
Food-web model of the studied systems. The classifications denote which role each species/food web component has in the tested statistical models (Table 1). Gray seal is the most abundant seal species in the Baltic Sea and feed mainly on sprat, herring and cod (Lundström et al. 2010). Cod is the predominant piscivorous fish in many parts of the region, feeding mainly on sprat and herring, which together constitute around 85% of the pelagic fish species in terms of biomass (Elmgren 1984). Cod also feeds on benthic invertebrates, in particular the isopod *Saduria entomon* (Zalachowski 1985). Sprat of all sizes are zooplanktivorous, whereas larger herring also feeds on benthic species (Casini et al. 2004). In particular, the lipid-rich amphipod *Monoporeia affinis* can constitute a large proportion of the herring diet (Aneer 1975). *S. entomon* feeds mainly on *M. affinis;* in the Bothnian Sea, they form a tightly coupled predator–prey system (Sparrevik and Leonardsson 1998). Polychaetes contribute to the diet of herring and *S. entomon* to a smaller extent (not shown in this figure)

The study focused on key consumers of the pelagic and benthic food-webs, encompassing species which are geographically widespread, contribute substantially to overall biomass (e.g. Elmgren 1984) and are adequately represented in monitoring data (Table 1, Table S1). The studied taxa are either predators, prey, or both, and all taxa feeding on the same prey are additionally potential competitors, including potential intra-specific competition (Fig. 2). With the exception of cod and sprat, all food-web components are abundant in both basins.

**Table 1:** Metrics on physiological status and population/community traits indicative of food value to consumers. “Sample size” gives the range (median within brackets) of number of individuals analyzed per year for seal blubber thickness, fish condition, weight-at-age, fat content, and *Monoporeia* viable embryos, and number of samples per year for population data on *Saduria* and zooplankton community data. In addition to the metrics listed, data on prey abundance/biomass were included in the PLS regressions (Table S1). The last column lists all potential explanatory variables assessed, for metrics used as response variables in these models. See Fig. 1 for location of sampling stations, and Table S1 for more detailed specifications of the data used. BP denotes Baltic Proper and BoS denotes Bothnian Sea

Food-web component	Status metric	Description of metric (unit)	Spatial delineation	Sample size	Rationale for use	Potential predictors (see also Table 3)
Used only as response variable						
Gray seal	Blubber thickness (BT)	Blubber layer (mm), in males, ages 4–20, bycatch	Whole Baltic Sea (Baltic Proper + Bothnian Sea)	1–10 (5)	Indicator of nutritional status in seals (HELCOM 2018)	Abundance of prey: cod, herring, sprat Prey quality: cod, herring, sprat Competitors: seal, cod Predators: NA
Used as response variable or as predictor						
Cod	Condition (c)	Calculated (Eq. 1, g/cm^b^) in 30–40 cm fish	Baltic Proper	162–2121 (811)	Physiological status indicator, indicator of fish well-being. May respond to both food availability and quality. Could potentially be affected also by predation if weaker individuals are easier to catch	Abundance of prey: herring, sprat, Saduria Prey quality: herring, sprat, Saduria Competitors: seal, cod Predators: seal
	Fat content	In liver (%) In 30 cm fish	Baltic Proper	10–20	Higher fat content usually results from higher food quality. Could potentially also be affected by selective predation	
Herring	Weight-at-age (WAA)	Ages 3–5 (gram)	Baltic Proper + Bothnian Sea	BP: 70–544 (327) BoS: 33–477 (53)	Body size (weight); may respond to both food availability and quality, but also size-selective predation	Abundance of prey: zooplankton, Amphipods or Amphipods + Polychaete Prey quality: zooplankton Competitors: sprat, herring, Saduria Predators: seal, cod (no benthic prey variables for BP WAA or C, see text for details)
	Condition (c)	Calculated (Eq. 1, g/cm^b^) in 18 cm fish	Baltic Proper + Bothnian Sea	BP: 70–544 (327) BoS: 33–477 (53)	See cod	
	Fat content	In muscle (%) In 15–20 cm fish	Baltic Proper + Bothnian Sea	20 (BP) 24 (BoS	See cod	
Sprat	Weight-at-age (WAA)	Ages 2–4 (gram)	Baltic Proper	34–475 (236)	See herring	Abundance of prey: zooplankton Prey quality: zooplankton Competitors: herring, sprat Predators: seal, cod
	Condition (c)	Calculated (Eq. 1, g/cm^b^) in 12 cm fish	Baltic Proper	34–475 (236)	See cod	
*Saduria entomon*	Mean body weight (mw)	Population trait: ratio total population biomass and abundance	Baltic Proper + Bothnian Sea	BP: 6–14 (10) BoS: 2–33 (20)	Larger *Saduria* are preferentially eaten by cod (Casini unpublished). Mean size could also reflect changes in population structure by increased recruitment alternatively poorer growth	Abundance of prey: Amphopods or Amphipods + Polychaeta Prey quality: NA Competitors: herring, *Saduria* Predators: cod
Used only as predictor						
Zooplankton	Mean size (ms)	Community trait: Ratio total abundance and biomass	Baltic Proper + Bothnian Sea	BP: 6–8 BoS: 4–8	HELCOM core indicator. Mean size reflects the proportion of larger copepods and cladocerans in the zooplankton community, which are generally more profitable for herring and sprat compared to smaller taxa (Gorokhova et al. 2016)	NA
Used only in descriptive statistics						
*Monoporeia affinis*	Viable embryos (ve)	Population mean (number/female)	Baltic Proper + Bothnian Sea	BP: 12–324 (143) BoS: 74–963 (144)	HELCOM Supplementary indicator. Embryo viability responds also to contaminants in sediments (Sundelin and Eriksson 1998) hence it is not used as predictor in this study	NA

### Metrics and data used

Basin-specific data were used for zooplankton, benthic invertebrates and herring. Data for cod and sprat were only applied for the BP analysis, in agreement with their principal current natural distribution (ICES 2016). Gray seals are mobile and considered to comprise a single population in the Baltic Sea (Galatius et al. 2015) and was analyzed across BP and BoS combined.

The physiological status (estimated on individual level) or the population- and community-level traits (all referred to as food value) of each taxon was quantified by at least one metric in each assessed basin. The metrics typically represented variables covered by current environmental monitoring and assessment, and varied depending on taxon-specific properties and data availability (Table 1, Supplementary Tables S1, S2). In addition, abundance/biomass data for each taxon were used, as obtained from the Swedish National Marine Monitoring Program and international surveys (Table S2). Benthic invertebrate and zooplankton data were acquired from the SHARK database (www.smhi.se), except for open sea benthic data (Fig. 1) which were from the Finnish SYKE HERTA database (http://www.syke.fi), and fish data from ICES (www.ices.dk). Time series on zooplankton biomass (including copepods, cladocerans and rotifers) were integrated from national and international stations in coast and open sea (Gorokhova et al. 2016; Fig. 1).

Gray seal abundance was estimated based on surveys carried out during the peak of the molting period (May–June) by international monitoring coordinated by HELCOM (Galatius et al. 2015). Gray seal physiological status was based on the blubber thickness of adult males caught as incidental bycatch during autumn, a time of the year before the winter when the blubber thickness is expected to respond primarily to food availability (HELCOM 2018). Gray seal occur in the entire Baltic, but the population is centered in the archipelagos of Stockholm, Åland and Turku. Since gray seals are highly mobile and movements between basins occur frequently we did not separate data for seal blubber thickness or abundance for the different basins.

Abundance data for herring and sprat were obtained from analytical assessment models provided by ICES (2016), and the abundance of cod was estimated based on data from the Baltic International Trawl Survey (Casini et al. 2016; ICES 2016). For herring and sprat, data representing the whole population, as well as age groups 3–5 (herring) and 2–4 (sprat) years were included. For cod, data representing the whole population, as well as individuals larger than 30 cm (mature fish; ICES 2016) were included. For all fish species, physiological status was expressed based on the individual body condition;

1 $$ {\text{Individual condition}} = \frac{W}{{L^{\text{b}} }} $$

where *W* and *L* are the weight and the total length of the fish, respectively, and *b* is the slope of the overall Ln weight–Ln length relationship. For herring and sprat, the mean weigh-at-age was also used (WAA, data obtained from ICES 2016). Both metrics were estimated based on the Swedish part of the Baltic International Trawl Survey (for cod) and the Baltic International Acoustic Surveys (for sprat and herring), both performed in autumn (Casini et al. 2011, 2016; ICES 2016). In addition, data on the fat content of cod and herring from the Swedish national monitoring program were included (Table 1).

Benthic macrofauna was represented by the predatory isopod *Saduria entomon*, the deposit-feeding amphipods *Monoporeia affinis* and *Pontoporeia femorata,* and the polychaetes *Marenzelleria* spp., and *Bylgides sarsii*. *Saduria* is an important food for cod (Zalachowski 1985) while the lipid-rich amphipods, and, to some extent, polychaetes, are eaten by adult herring (Aneer 1975; Casini et al. 2004). In the northern BoS, *Bylgides* does not occur, whereas *Pontoporeia* occurs only sparsely. *Marenzelleria*, a recently introduced non-indigenous polychaete, became abundant in both basins in the past decade. The total abundance of *Monoporeia* and *Pontoporeia* (included as the variable “amphipods” in the analyses), and of amphipods together with polychaetes (variable “AmpPol”) were obtained from all stations in BoS and from the Askö-stations in BP. Open sea deep stations in the BP (Fig. 1) are frequently affected by hypoxia and lack permanent benthic macrofauna since 2000 (Villnäs and Norkko 2011). Hence, for these stations, only the frequency of occurrence (%) of (the migratory) *Saduria* was used, and compared to *Saduria* frequency of occurrence from the other regions. To represent its potential food value for cod, the mean weight of *Saduria* (mw, Table 1) was calculated from data on population abundance and biomass (i.e. this metric represented a population trait rather than individual physiological status) from the Askö- and the N-stations (Fig. 1). To avoid dependency (and autocorrelation) with mw, *Saduria* abundance was represented by frequency of occurrence also at coastal stations. For *Monoporeia,* physiological status was based on the number of viable embryos (ve) per ovigerous female (Sundelin and Wiklund 1998), based on five stations per basin for which long-term data were available (Table 1, Tables S1, S2).

Zooplankton biomass and mean size were based on average monthly abundance and biomass values for June–September (Fig. 1, Supplementary Table S1). We calculated the average summer biomass (mg m^3^) and mean zooplankter size (µm ind^−1^) as described in Gorokhova et al. (2016). Zooplankton mean body size was used as a metric to represent the prey food value for zooplanktivores (herring and sprat). In the Baltic zooplankton communities, the mean size reflects the proportion of larger copepods and cladocerans (i.e., a community characteristic) which are generally more profitable prey items to herring than small-bodied cladocerans, nauplii and rotifers (Flinkman et al. 1998; Casini et al. 2004). Together, total zooplankton biomass and mean zooplankter size represent food availability and food value for zooplanktivorous fish in the area (Gorokhova et al. 2016).

### Data treatment prior to analyses

All variables were normalized (zero mean, unit variance) using the long-term (22 years) mean and standard deviation values, to focus on the changes in relative rather than in absolute values, and to avoid ordination analyses to be driven by variables with largest values. Abundance data were square-root transformed before normalization. For *Monoporeia* ve, missing data for the first year (1993) were replaced with the zero score mean (0). For seal blubber thickness, missing values in 1993 and 1999 were replaced by a moving average of the preceding and proceeding 2 years, based on observations on a longer national data series (HELCOM 2018), which show many years of stable blubber thickness during the 1980s and a shift around 1994 towards decreasing values.

### Data analyses

#### Changes over time in physiological status and food-web structure

Directional trends in the physiological status/food value metrics as well as for abundance data over time were assessed by the non-parametric Mann–Kendall test. To identify any common changes over time in the studied status variables across species/groups or trophic levels, and years of high similarity, we applied a principal components analysis (PCA) on the normalized data. PCAs were performed separately for metrics reflecting physiological status/food value and abundances, and separately for each basin. Sprat and cod were only included for the BP. Using the same data sets, the level of similarity between adjacent years was assessed by Chronological clustering as implemented in Brodgar 2.7.4 linked to R3.3 (Highland statistics). Similarities among years were assessed based on Euclidean distances in all cases.

#### Explaining consumers’ physiological status

We predicted that high prey availability and high physiological status/food value (i.e., the energetic content) would have a positive influence on the physiological status of consumers (predictions i and ii), while higher abundances of competitors would have negative effects (iii), and predation may have positive or negative effects on the physiological status of prey (iv). The relationships of each physiological status metric (Table 1, in total 13 models) to the food-web structure (Fig. 2, Table 1) and to prey food value (Table 1) were assessed using Partial Least Square Regression (PLSR) analyses (Wold et al. 2001). The choice of method was motivated by the characteristics of the data set, encompassing relatively short time series (22 years) and many potential explanatory variables. PLSR is a generalization of multiple linear regression that is particularly well suited for analyzing data sets where the number of observations per variable is relatively low compared to the number of explored variables (Wold et al. 2001; see details on cross-validation procedure below). PLSR is also suited for dealing with potentially collinear predictors, allowing even for correlated explanatory variables to be included. Another benefit of the PLSR approach in the context of our research questions and the data structure, is that the model evaluation is based on optimization of the explanatory and predictive capacity of the model.

The models were fitted separately for each of the response metrics (Table 1). Between 3 and 12 potential explanatory variables were used in each model, representing the abundance of potential predators and prey, or the physiological/food value of prey, as well as potential competitors for prey (predictions i–iv, Fig. 2, Table 1). For the fish species, several measures of physiological status were included (Table 2) to compare model outcomes in relation to the tested predictors. Gray seal blubber thickness, which was compiled at the pan-Baltic scale, was regressed against variables representing both BP and BoS. Sprat and cod were only regressed against BP variables since they are more abundant there. All other metrics for the other taxa were related to the basin-specific variables. The benthic data were included in different formats depending on the explored response variables. For modeling BoS herring condition and WAA, a grand mean of Amp, or AmpPol, from all stations in BoS was used as a potential explanatory variable. However, for the modeling of BoS herring fat content, benthic data were taken only from station SR5, as the herring fat content data originated from this area (Fig. 1). BP herring fat content was monitored close to Askö (Fig. 1), and, hence, was related to benthic variables from this area. We avoided extrapolating the same coastal benthos data to models on BP herring condition and WAA, representing herring at the scale of the whole basin of BP, due to differences in spatial coverage. Since the variables Amp and AmpPol were autocorrelated, we tested them separately and the variable contributing to the better model was subsequently chosen. In addition, *Saduria* mw was estimated based on individuals sampled from coastal stations, and, hence, explanatory variables representing its prey were restricted to the Askö or the N-cluster stations (Fig. 1).

**Table 2:** Status metrics used as response variables in the partial least square regressions (PLSR) with model evaluation parameters, sorted by species and Basin. *R*^2^*X* = the explained variance of predictors by each PLSR component; *R*^2^*Y* = the explained variance of dependent variables by each PLSR component (analogous to the coefficient of determination *R*^2^ in regression analysis); and *R*^2^*Q* = model prediction capacity. See Table 3 for details on the models outputs. BoS denote Bothnian Sea, BP Baltic Proper and p-B pan-Baltic. The assessed physiological status or population/community traits variables are: mean weight (mw), condition (c), weight-at-age (WAA), fat (%) and blubber thickness (BT) (see Table 1 for details)

Species	*Saduria*		Sprat		Herring						Cod		Seal
Basin	BoS	BP	BP		BoS			BP			BP		p-B
Status metric	mw		WAA	c	WAA	c	Fat	WAA	c	Fat	c	Fat	BT
*R*^2^*X*	0.72	0.56	0.69	0.57	0.65	0.73	0.77	0.54	0.78	0.58	0.8	0.63	0.81
*R*^2^*Y*	0.55	0.35	0.70	0.70	0.47	0.42	0.41	0.8	0.66	0.5	0.8	0.49	0.32
*R*^2^*Q*	0.5	0.3	0.42	0.57	0.44	0.35	0.37	0.78	0.61	0.45	0.7	0.41	0.17
Components (nr)	1	1	2	1	1	1	1	1	1	1	1	1	1
Eigenvalues	1.43	1.69	1.70; 0.87	1.70	1.93	1.43	1.53	1.08	1.55	1.71	1.58	1.25	2.41

The analyses were performed with the NIPALS (Nonlinear Iterative Partial Least Squares) algorithm, as implemented in STATISTICA 13 (StatSoft, Inc. 2017). The models were validated based on the obtained values of *R*^2^*Y*, which is analogous to the coefficient of determination (*R*^2^) used in the regression analysis; and of R^2^Q, which represents the model’s predictive capacity. Model evaluation followed Lundstedt et al. (1998) on that a biological PLSR model is of good quality when *R*^2^*Y* > 0.7, and *R*^2^*Q* > 0.4. Variable selection was performed based on the VIP scores (variable importance for projection), which is the weighted sum of squares of the PLSR weights. All potentially relevant diet variables were initially included (Fig. 2, Table 2), and variables with VIP scores > 0.7 were used further in the model selection (Kaddurah-Daouk et al. 2011). Thereafter, the potential effects of competitors and predators were assessed in the same way; all variables that maximized *R*^2^*Y* and *R*^2^*Q* were retained in the final model. The number of variables in the final model was identified following the V-fold cross-validation. The autocorrelation of model residuals was evaluated using the ARIMA algorithm (Statsoft, Inc. 2017). Where significant 1-year lags were detected, the model was rerun, including the lagged year response variable as an additional predictor variable, and residuals were again checked for partial autocorrelation. No further action was required to account for autocorrelation in any of the models. Because all models but one were best explained by a single PLS component, we also fitted linear models with single predictors. Among these we identified best models based on Akaike information criteria (AIC) estimates, and compared the predictors identified using this approach with those from the PLSR approach.

## Results

### Changes in physiological status and food-web structure

**Fig. 3:**
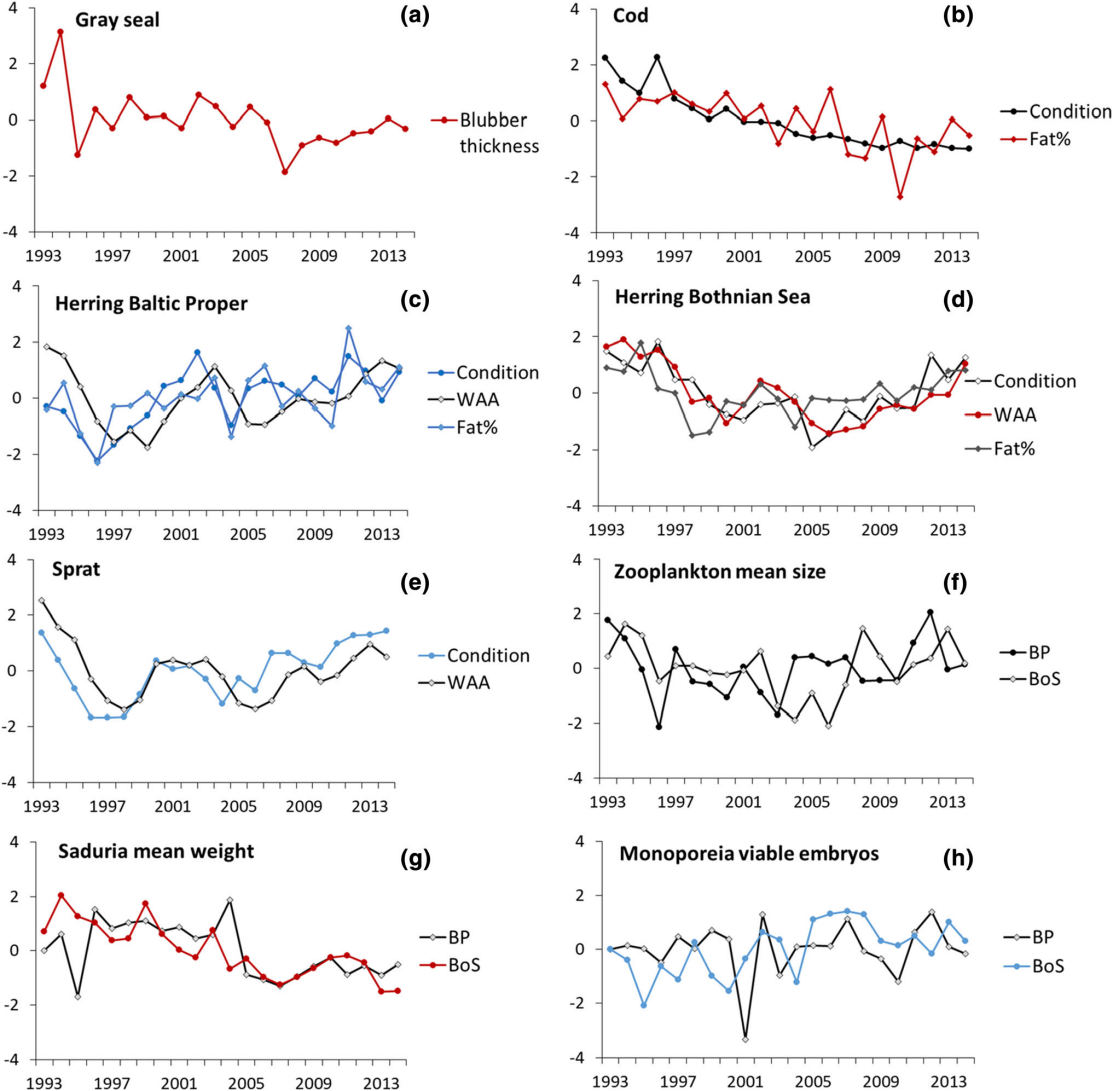
Temporal development of the physiological status metrics (seal, cod, herring, sprat and *Monoporeia*) and population/community traits (*Saduria*/zooplankton). Values show normalized data to aid comparisons. WAA denotes weight-at-age. Red = decreasing over time, blue = increasing, black = no change over time, based on Mann–Kendall test (*p* < 0.05). See also Fig S1 for analyses of common trends within each basin (PCA), and text and Table 1 for description of metrics

**Fig. 4:**
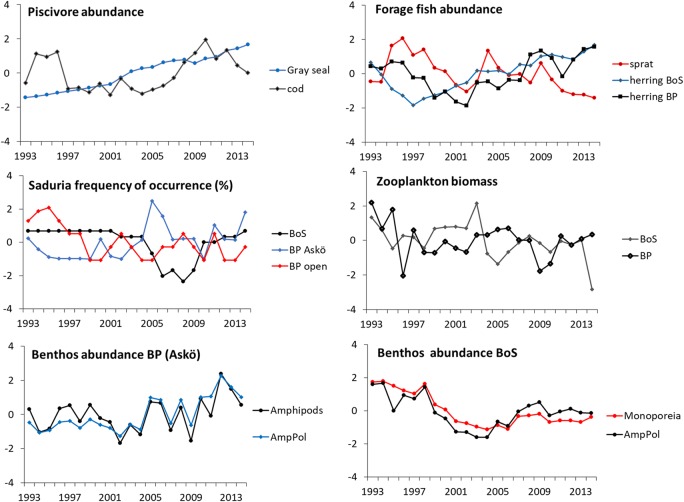
Temporal trends in the abundance, biomass or frequency of occurrence (%) of the species or species groups used as predictors in the PLS regressions. Values show normalized data to aid comparisons. Herring and sprat abundances show sums for all size classes. AmpPol represents the total sum of amphipods and polychaetes (hence, correlated with Amphipods). Red = decreasing over time, blue = increasing, black = no change over time based on Mann–Kendall test (*p* < 0.05, detailed results in Table S3. See also Fig S1 for analyses of common trends within each basin (PCA), and text and Table 1 for description of metrics

There were long-term trends in status metrics of most consumers in both basins (Fig. 3; Table S3). The physiological status of seal and of cod decreased over the studied time period (Fig. 3a, b), whereas those of sprat and herring generally increased, at least over the later decade (Fig 3c–e). For invertebrates, trends in *Monoporeia* viable embryos and *Saduria* mean weight differed between basins (Fig. 3g, h), while zooplankton mean size had no unidirectional trend. The PCA analyses showed that fish metrics representing the same species and basins were generally correlated with each other (Supplementary Fig. S1). For the physiological status/food value metrics, there was no unidirectional change over time among different trophic levels in any of the two basins, (Supplementary Fig. S1). The temporal trends in abundance/biomass metrics found for a number of species (Fig. 4; Table S3) were also partly reflected in the PCA for the BoS. In both sub-basins, these analyses show a shift between the earlier years studied (until years between 1996 and 1998 for the different plots) for both physiological status/food value and abundances, reflecting a decreasing physiological status/food value and changes in the relative abundance of taxa from different trophic levels.

### Explaining consumers’ physiological status

**Fig. 5:**
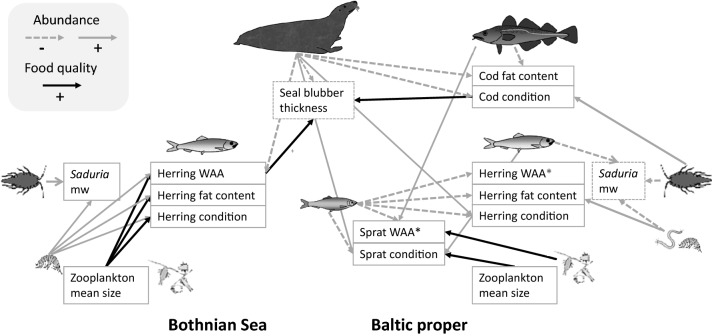
Summary of the model results for the Bothnian Sea and the Baltic proper. Arrows illustrate significant links according to the PLSR models (See Table 3), and point in the direction from predictor to response variables. Gray arrows denote abundances of prey, competitors or predators (dashed = negative, whole gray = positive association) and black arrows denote food quality aspects (always positive association). *mw* mean weight, *WAA* weight-at-age. See Table 3 for lag effect results, which were found for herring and sprat WAA in the BP. Note also that arrows pointing to gray seal blubber thickness and *Saduria* mean weight in the BP are included for completeness, but those models had a predictive capacity and proportion explained below the criteria (Table 2)

Models meeting the evaluation criteria were obtained for 11 of the 13 physiological/food value metrics tested (Table 2; exceptions were models for gray seal blubber thickness and *Saduria* mean weight in the BP). Only the BP herring and sprat WAA models resulted in including lagged values due to the significant autocorrelation. In line with our predictions, the changes in the physiological status of consumers were often explained by a combination of responses, i.e., a positive relation to the prey abundance (prediction i) and to the physiological/food value of prey (prediction ii), a negative relation to the abundance of competitors (prediction iii), and a negative or positive relation to predators (prediction iv). Only two of 28 cases showed a direction of association that did not follow our predictions (herring had a positive effect on sprat condition, and AmpPol a negative effect on *Saduria* mw in the BP). The results are described below, presented in detail in Table 3 and illustrated in Fig. 5. Generally, results from linear model results selected based on AIC were largely similar to PLSR results, although cod and gray seal models included additional predictors, i.e. herring and sprat WAA (Table S4).

**Table 3:**
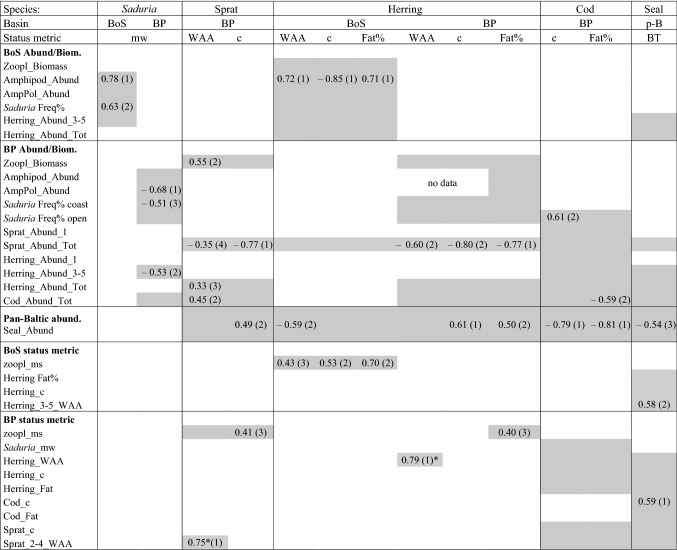
Partial least square regression (PLSR) model results. *Saduria* and herring were assessed in both Basins (Baltic Proper, BP, and Bothnian Sea, BoS), cod and sprat in BP only, and gray seal as a pan-Baltic (p-B) population. The predictors represent stocks (abundance, biomass or frequency of occurrence) potentially affecting prey availability, competition and/or predation (Fig. 1, text for details), as well as physiological status or population/community traits of relevant prey (all representing food value to consumers), and are listed in column 1. Each column represents a model and predictors entering the model are highlighted in gray. Values are shown for variables with a Variable of Importance (VIP) score above 0.7 and which improve the model predictive capacity while maximizing *R*^2^*Y* (the explained variance of response variables by each PLSR component). The values are the X-loadings, which describe the association (positive or negative) with PLSR component 1. Numbers in brackets denote their ranking based on VIP score. The results are summarized in Fig. 5. The assessed physiological status or population/community traits variables are: mean weight (mw), condition (c) and weight-at-age (WAA) and fat (%) and blubber thickness (BT); see Table 1 for details. *denotes that lagged values of the response variable are included in the model

### Bottom-up control (predictions i and ii)

In BoS, the herring physiological status and *Saduria* mean weight were explained by the amphipod abundance as well as zooplankton mean size. In BP, the mean size of zooplankton contributed to explaining both physiological status in sprat and herring fat content. *Saduria* frequency of occurrence was a significant positive predictor for cod condition. Zooplankton biomass was only a significant predictor for sprat WAA.

### Competition (prediction iii)

In the BP, the abundance of competitors was included in many of the models. All herring physiological status metrics were negatively related to the sprat abundance, and *Saduria* mean weight was negatively related to herring abundance (competitors for benthic prey). Moreover, positive association was detected between herring abundance and sprat condition. Intra-specific competition was indicated by the models for sprat condition and WAA, cod fat content, and gray seal blubber thickness. In the BoS, positive associations were seen between *Saduria* mean weight and frequency of occurrence.

### Top-down control (prediction iv)

In the BP, a positive association was found between gray seal abundance and the condition of sprat and herring as well as herring fat content. In contrast, gray seal abundance had a negative effect on BoS herring WAA. Fat content and condition of cod was negatively associated to gray seal abundance in the best PLSR model, whereas it was additionally explained by sprat WAA and herring WAA in the best linear model based on AIC (i.e. bottom-up). Further, sprat WAA was positively related to cod abundance.

## Discussion

We show that both top-down and bottom-up effects control physiological status of consumers across multiple levels in Baltic Sea food-webs (Fig. 5). During the study period, the physiological status declined in the piscivores (gray seals, cod), whereas it increased—at least during the last decade—for their main prey, the mesopredators herring and sprat. Trends in the physiological status or population/community characteristics of invertebrates were absent or basin-specific. The physiological statuses of cod, herring and sprat were influenced by a combination of prey availability, abundance of competitors and predators; herring and sprat status were also influenced by prey size.

The availability of prey is important for the physiological status of the consumers (prediction i) as shown for herring and *Saduria* in the Bothnian Sea and for cod (condition only) in the Baltic Proper. All three metrics on the physiological status of Bothnian Sea herring were strongly linked to variations in the abundance of the amphipods (i.e. *Monoporeia*), which are a lipid-rich food source (Hill et al. 1992). With respect to zooplankton as prey for herring and sprat, the prey food value, assessed here as mean size of a zooplankter in the community, was more important than the total zooplankton biomass (prediction ii). In zooplankton, the mean size incorporates the contribution of large lipid-rich copepods and cladocerans to total zooplankton biomass, which are important prey for herring condition and growth (Flinkman et al. 1998; Casini et al. 2004; Östman et al. 2014). Changes in the food value of lower consumers (e.g. benthic prey) can cascade upwards (e.g. to herring WAA) and affect the physiological status of the top consumers (gray seal blubber thickness). Although our model on blubber thickness had a low predictive capacity, the link between herring WAA and gray seals have been demonstrated by Kauhala et al. (2017). Decreased WAA of older herring in the Baltic Sea has been related to decrease in the population size of mysid shrimps (Kostrichkina 1982). Our study further highlights the importance of deposit-feeding amphipods for the physiological status of herring.

A decreased mean weight of *Saduria*, which feeds mainly on *Monoporeia*, was also related to the decline in the amphipod abundance in the Bothnian Sea, also likely leading to additional negative effects on *Saduria* population size. Populations of *Monoporeia* collapsed in the Bothnian Sea in the early 2000s, presumably because of deteriorated feeding conditions due to extreme precipitation and runoff (Eriksson-Wiklund and Andersson 2014). Despite higher reproductive success in the recent years, the *Monoporeia* population abundance remains low, suggesting that the increasing in abundance of herring may exert some top-down control. The non-indigenous species *Marenzelleria* was not included or positively associated to consumer status in any of the models suggesting that it cannot replace *Monoporeia* as prey for higher trophic levels.

Support for the importance of benthic prey availability was also found for cod in the Baltic proper. The deteriorating cod condition was linked to the decreasing frequency of occurrence of *Saduria* in the open Baltic proper. *Saduria* are prey items for cod and also contain high levels of essential fatty acids, which can be complementary to the fat composition in forage fish that cod eat (Røjbek et al. 2014). Casini et al. (2016) hypothesised a link between hypoxia-related decrease in benthic prey and cod condition, but had no data on benthic prey. Our results support this hypothesis and suggest a mechanistic explanation. We found that *Saduria* populations in the benthos of the open sea have declined, likely due to benthic hypoxia (Villnäs and Norkko 2011), and decline in benthos was related to cod condition. However, this decline is not measured in the coastal area (Askö), where increases occurred, but *Saduria* mean weight declined. This pattern could be the result of hypoxia-induced migrations of *Saduria* to the more oxygenated coastal areas, increased competition and, consequently, decreasing mean weight in the coastal *Saduria* populations.

The declines in cod condition and fat content were best explained by the increased abundance of gray seals, suggesting competition for prey (herring and sprat) between gray seals and cod (prediction iii), or selective feeding by gray seals on cod in good condition (prediction iv, Kohl et al. 2015). Alternative explanations could be related to increased parasite infestation in cod, enhanced by gray seals which are the final host (Horbowy et al. 2016), or the correlation merely representing the general decreasing trend in cod physiological status (coinciding with linear increase in gray seal abundance, Fig. 3a). Despite the relatively low abundance of cod compared to the historical levels (ICES 2016), we found indications of intra-specific competition (prediction iii, as found also by Casini et al. 2016). In the Baltic Proper, it is likely that the spatial mismatch between cod and sprat (Casini et al. 2011) and the hypoxia-related reductions in benthic prey would result in intra-specific competition for food. The best models based on AIC (Table S4) suggest that body size of herring and sprat have additionally contributed to explain the declining condition and fat in cod. Size of fish prey has previously been linked also to cod growth, and the lack of suitably sized prey (herring and sprat) for piscivorous cod was suggested to contribute to the lack of cod recovery (Gårdmark et al. 2015).

The increasing physiological status (i.e. condition and fat content) of herring and sprat in the Baltic Proper and also in the last decade in the Bothnian Sea has not previously been reported. However, both the WAA and condition of herring are still at historically low levels (e.g. Casini et al. 2010). The physiological status of herring in the Baltic proper was mainly negatively related to the abundance of sprat, indicating that the interspecific competition (prediction iii, Casini et al. 2010) continues to be important. It also suggests an asymmetrical interaction since sprat condition was positively associated to herring abundance.

The physiological status of mesopredators in the Baltic Proper was also positively associated with gray seal abundance, with respect to condition (herring) and fat content (sprat). This could also indicate a positive effect of predation (prediction iv) if prey in poorer condition are preferred or, alternatively, easier to catch. However, predation could result in reduced intra-guild competition and compensatory growth (Casini et al. 2010, 2011). Interestingly, the condition and WAA of sprat were related to gray seal and cod abundances, respectively, suggesting that these top consumers partition resources to some extent (cod preying on small individuals and gray seal on individuals in bad condition).

Finally, it should be noted that this study did not attempt to test the relationships between changes in environmental conditions and the physiological status in piscivores, mesopredators or food value in invertebrates. Lower salinity (as well as increasing herring population size) has been associated to reduced lipid content in Baltic herring during the same time period as studied here, due to the more energetically costly osmoregulation with decreasing salinities (Rajasilta et al. 2019). Casini et al. (2016) discuss potential negative effects on low oxygen concentrations for physiological status in cod, which is also a factor relevant for the benthic invertebrate *Saduria*, and an increasing environmental concern in the Baltic Sea (Carstensen et al. 2014). Warmer temperature will likely improve growth conditions for both herring and sprat as long as food is not limited (Margonski et al. 2010), but will unlikely affect fish lipid content or the blubber thickness in gray seals which spend most time at greater depths, where temperature is more constant. In addition, both fish fat and seal blubber is measured in autumn before any potential effects of the colder winter months would be seen.

## Conclusions

Our study highlights the importance of food value as well as quantity of prey for population-level changes in the physiological status of consumers in the Baltic Sea. It also highlights the significance of benthic prey for the condition of fish in both basins, in addition to the food value of zooplankton prey, and inter- and intra-specific competition. The importance of benthic invertebrates for pelagic top consumers is often neglected in multi-species models (but see e.g. Niiranen et al. 2012; Huss et al. 2014) and in the management of commercially important fish species (ICES 2016). Benthic population stocks may decrease in the future due to continuously decreasing oxygen conditions in the deep water of the Baltic Proper related to eutrophication and climate change, and attributed to climate-related brownification in the Bothnian Bay (Andersson et al. 2015). Our results suggest that changes in the benthos and zooplankton communities will likely continuously affect the physiological status in the higher trophic levels, including the weight and condition of commercially exploited fish species. Hence, we highlight the importance for fisheries and environmental management to take account of species interactions across trophic levels in the food-web. Under this approach, the key parameters for monitoring performance should include not only population size reflecting the food-web structure, but also the physiological status of the prey and predators. Many of the physiological status metrics studied here are already included in the Baltic Sea monitoring and assessment programs, but their integrated use in food-web analyses is not yet developed; the latter is essential for meeting current management challenges.

## Electronic supplementary material

Below is the link to the electronic supplementary material.

Supplementary material 1 (PDF 456 kb)

## References

[CR1] Andersson A, Meier HEM, Ripszam M, Rowe O, Wikner J, Haglund P, Eilola K, Legrand C (2015). Projected future climate change and Baltic Sea ecosystem management. Ambio.

[CR2] Aneer G (1975). Composition of food of the Baltic herring (*Clupea harengus* v. membras L.), fourhorn sculpin (*Myoxocephalus quadricornis* L.) and eel-pout (*Zoarces viviparous* L.) from deep soft bottom trawling in the Askö-Landsort area during two consecutive years. Merentutkimuslait H. Julk./Havsforskningsinstitutets skrift.

[CR3] Bogstad B, Gjøsæter H, Haug T, Lindstrøm U (2015). A review of the battle for food in the Barents Sea: Cod vs. marine mammals. Frontiers in Ecology and Evolution.

[CR4] Carstensen J, Andersen JH, Gustafsson BG, Conley DJ (2014). Deoxygenation of the Baltic Sea during the last century. Proceedings of the National Academy of Science of the United States of America..

[CR5] Casini M, Cardinale M, Arrhenius F (2004). Feeding preferences of herring (*Clupea harengus*) and sprat (*Sprattus sprattus*) in the southern Baltic Sea. ICES Journal of Marine Science.

[CR6] Casini M, Lövgren J, Hjelm J, Cardinale M, Molinero JC, Kornilovs G (2008). Multi-level trophic cascades in a heavily exploited open marine ecosystem. Proceedings of the Royal Society B Biological Sciences.

[CR7] Casini M, Bartolino V, Moliniero JC, Kornilovs G (2010). Linking fisheries, trophic interactions and climate: Threshold dynamics drive herring *Clupea harengus* growth in the central Baltic Sea. Marine Ecology Progress Series.

[CR8] Casini M, Kornilovs G, Cardinale M, Möllmann M, Grygiel W, Jonsson P, Raid T, Flinkman J (2011). Spatial and temporal density-dependence regulates the condition of central Baltic Sea clupeids: Compelling evidence using an extensive international acoustic survey. Population Ecology.

[CR9] Casini M, Käll F, Hansson M, Plikshs M, Baranova T, Karlsson O, Lundström K, Neuenfeldt S (2016). Hypoxic areas, density-dependence and food limitation drive the body condition of a heavily exploited marine fish predator. Royal Society Open Science.

[CR10] Elmgren R (1984). Trophic dynamics in the enclosed, brackish Baltic Sea. Rapports et Procès-verbaux des Reunions. Conseil International pour l Exploration de la Mer.

[CR11] Elmgren R, Hill C, Ormond RFG, Gage JD, Angel MV (1997). Ecosystem function at low biodiversity—the Baltic example. Marine biodiversity, patterns and processes.

[CR12] Elmgren R, Blenckner T, Andersson A (2015). Baltic Sea management: Success and failures. Ambio.

[CR13] Eriksson-Wiklund A-K, Andersson A (2014). Benthic competition and population dynamics of *Monoporeia affinis* and *Marenzelleria* sp. in the northern Baltic Sea. Estuarine, Coastal and Shelf Science.

[CR14] Flinkman J, Aro E, Vuorinen I, Viitasalo M (1998). Changes in northern Baltic zooplankton and herring nutrition from 1980s to 1990s: Top-down and bottom-up processes at work. Marine Ecology Progress Serie, s.

[CR15] Galatius, A., M. Ahola, T. Härkönen, I. Jüssi M. Jüssi, O. Karlsson, and M. Verevkin. 2015. Guidelines for seal abundance monitoring in the HELCOM area 2014. Updated 11 Feb 2015. http://helcom.fi/action-areas/monitoring-and-assessment/manuals-and-guidelines/seal-abundance-guidelines.

[CR16] Gårdmark A, Casini M, Huss M, Van Leeuwen A, Hjelm J, Persson L, de Roos AM (2015). Regime shifts in exploited marine food-webs: Detecting mechanisms underlying alternative stable states using size-structured community dynamics theory. Philosophical Transactions Royal Society, Series B.

[CR17] Gorokhova E, Lehtiniemi M, Postel L, Rubene G, Amid C, Lesutiene J, Uusitalo L, Strake S (2016). Indicator properties of Baltic zooplankton for classification of environmental status within Marine Strategy Framework Directive. PLoS ONE.

[CR18] Harding KC, Fujiwara M, Härkönen T, Axberg Y (2005). Mass dependent energetics and survival in harbour seal pups. Functional Ecology.

[CR19] Harwood LA, Smith TG, George JC, Sandstrom SJ, Walkusz W, Divoky GJ (2015). Change in the Beaufort Sea ecosystem: Diverging trends in body condition and/or production in five marine vertebrate species. Progress in Oceanography.

[CR20] HELCOM, 2018. Nutritional status of marine mammals. HELCOM core indicator report. Online. Viewed 2018-08.30. http://www.helcom.fi/Core%20Indicators/Nutritional%20status%20of%20seals%20HELCOM%20core%20indicator%202018.pdf.

[CR21] Hill C, Quigley MA, Cavaletto JF, Gordon W (1992). Seasonal changes in lipid content and composition in the benthic amphipods *Monoporeia affinis* and *Pontoporeia femorata*. Limnology and Oceanography.

[CR22] Horbowy J, Podolska M, Nadolna-Altyn K (2016). Increasing occurrence of anisakid nematodes in the liver of cod (*Gadus morhua*) from the Baltic Sea: Does infection affect the condition and mortality of fish?. Fisheries Research.

[CR23] Huss M, de Roos AM, van Leeuwen A, Gårdmark A (2014). Facilitation of fisheries by natural predators depends on life history of shared prey. Oikos.

[CR24] ICES, 2016. Report of the Baltic Fisheries Assessment Working Group (WGBFAS), 12-19 April 2016, ICES HQ, Copenhagen, Denmark. ICES CM 2016/ACOM:11. 594 pp. http://www.ices.dk/sites/pub/Publication%20Reports/Expert%20Group%20Report/acom/2016/WGBFAS/01%20WGBFAS%20Report%202016.pdf.

[CR25] Kadin M, Österblom H, Hentati-Sundberg J, Olsson O (2012). Contrasting effects of food quality and quantity on a marine top predator. Marine Ecology Progress Series.

[CR26] Kaddurah-Daouk R, Boyle SH, Matson W, Sharma S, Matson S, Zhu H, Bogdanov MB, Churchill E (2011). Pretreatment metabotype as a predictor of response to sertraline or placebo in depressed outpatients: A proof of concept. Translational Psychiatry.

[CR27] Kauhala K, Bäcklin B-M, Raitaniemi J, Harding KC (2017). The effect of prey quality and ice conditions on the nutritional status of Baltic gray seals of different age groups. Mammal Research.

[CR28] Kohl KD, Coogan SCP, Raubenheimer D (2015). Do wild carnivores forage for prey or for nutrients? Evidence for nutrient-specific foraging in vertebrate predators. BioEssays.

[CR29] Kostrichkina EM (1982). Long-term dynamics of herring growth in the Baltic Sea in relation to oceanographic conditions and food availability. Fischerei Forschung: Wissenschaftliche Schriftenreihe.

[CR30] Laine P, Rajasilta M (1999). The hatching success of Baltic herring eggs and its relation to female condition. Journal of Experimental Marine Biology and Ecology.

[CR31] Lundstedt T, Seifert E, Abramo L, Thelin B, Nyström Å, Pettersen J, Bergman R (1998). Experimental design and optimization. Chemometrics and Intelligent Laboratory Systems.

[CR32] Lundström K, Hjerne O, Lunneryd S-G, Karlsson O (2010). Understanding the diet composition of marine mammals: Grey seals (*Halichoerus grypus*) in the Baltic Sea. ICES Journal of Marine Science.

[CR33] Margonski P, Hansson S, Tomczak MT, Grzebielec R (2010). Climate influence on Baltic cod, sprat, and herring stock–recruitment relationships. Progress in Oceanography.

[CR34] Marshall CT, Yaragina NA, Adlandsvik B, Dolgov AV (2000). Reconstructing the stock-recruit relationship for Northeast Arctic cod using a bioenergetic index of reproductive potential. Canadian Journal of Fisheries and Aquatic Sciences.

[CR35] Malzahn AM, Aberle N, Clemmesen C, Boersma M (2007). Nutrient limitation of primary producers affects planktivorous fish condition. Limnology and Oceanography.

[CR36] Niiranen S, Blenckner T, Hjerne O, Tomczak MT (2012). Uncertainties in a Baltic Sea food-web model reveal challenges for future projections. Ambio.

[CR37] Österblom H, Olsson O, Blenckner T, Furness RW (2008). Junk-food in marine ecosystems. Oikos.

[CR38] Östman Ö, Karlsson O, Pönni J, Kaljuste O, Aho T, Gårdmark A (2014). Relative contributions of evolutionary and ecological dynamics to body size and life-history changes of herring (*Clupea harengus*) in the Bothnian Sea. Evolutionary Ecology Research.

[CR39] Peters J, Diekmann R, Clemmesen C, Hagen W (2015). Lipids as a proxy for larval starvation and feeding condition in small pelagic fish: A field approach on match-mismatch effects on Baltic sprat. Marine Ecology Progress Series.

[CR40] Rajasilta M, Hänninen J, Laaksonen L, Laine P, Suomela J-P, Vuorinen I, Mäkinen K (2019). Influence of environmental conditions, population density and prey type on the lipid content in Baltic herring (*Clupea harengus membras*) from the northern Baltic Sea. Canadian Journal of Fisheries and Aquatic Science.

[CR41] Røjbek MC, Tomkiewicz J, Jacobsen C, Støttrup JG (2014). Forage fish quality: Seasonal lipid dynamics of herring (*Clupea harengus* L.) and sprat (*Sprattus sprattus* L.) in the Baltic Sea. ICES Journal of Marine Science.

[CR42] Sparrevik E, Leonardsson K (1998). Recruitment in the predacious isopod *Saduria entomon* (L.): Alternative prey reduces cannibalism. Journal of Experimental Marine Biology and Ecology.

[CR43] Sundelin B, Eriksson A-K (1998). Malformations in embryos of the deposit-feeding amphipod *Monoporeia affinis* in the Baltic Sea. Marine Ecology Progress Series.

[CR44] Taipale SJ, Kahilainen KK, Holtgrieven GW, Peltomaa ET (2018). Simulated eutrophication and browning alters zooplankton nutritional quality and determines juvenile fish growth and survival. Ecology & Evolution.

[CR45] Trites AW, Donelly CP (2003). The decline of Steller sea lions in Alaska: A review of the nutritional stress hypothesis. Mammal Review.

[CR46] Vainikka A, Mollet F, Casini M, Gårdmark A (2009). Spatial variation in growth, condition and maturation reaction norms of the Baltic herring (*Clupea harengus membras*). Marine Ecology Progress Series.

[CR47] Villnäs A, Norkko A (2011). Benthic diversity gradients and shifting baselines: Implications for assessing environmental status. Ecological Applications.

[CR48] Wold S, Sjöström M, Eriksson L (2001). PLS-regression: A basic tool of chemometrics. Chemometrics and Intelligent Laboratory Systems.

[CR49] Zalachowski W (1985). Amount and composition of food of cod (*Gadus morhua*) in the Southern Baltic in 1977–1982. Acta Ichthyologica Et Piscatoria.

