# Supplementary material for: Linking consumer physiological status to food-web structure and prey food value in the Baltic Sea
Source: Ambio. 2019 Jun 5;49(2):391–406. doi: 10.1007/s13280-019-01201-1 (PMC6965491; doi:10.1007/s13280-019-01201-1)
Supplement: Supplementary file 1 — Supplementary material 1 (PDF 456 kb) [file 13280_2019_1201_MOESM1_ESM.pdf]

Ambio

Electronic Supplementary Material
This supplementary material has not been peer-reviewed

Title: **Linking consumer physiological status to food-web structure and prey food value in the Baltic Sea**

Authors: Agnes M. L. Karlson, Elena Gorokhova, Anna Gårdmark, Zeynep Pekcan-Hekim, Michele Casini, Jan Albertsson, Brita Sundelin, Olle Karlsson, Lena Bergström

Supplementary table S1. Abundance, biomass or freq. of occurrence and metrics for physiological status or population/community trait (representing food value of prey to consumers) of studied species/food web components in the Baltic Sea. All data are for years 1993-2014. Abbreviations: SMNH – Swedish Museum of Natural History, IVL – Swedish Environmental Research Institute, SLU- Swedish University of Agricultural Research (Dept. Aquatic resources), SD – ICES (International Council for Exploration of the Sea) subdivisions (Fig. 1). Amphipods: *Monoporeia* and *Pontoporeia*, Polychaetes: *Marenzelleria* and *Bylgides.* See Fig 1 for location of sampling stations; details can be found in text and in supplementary Table S2.

**Food web component**	**Status metric**	**Status metric classification**	**Sampling status metric**	**Sampling Abundance / Biomass /Frequency of occurrence**	**Sampling Location (status metric and abund./biomass/Freq.)**	**Data holder**
Grey seal	Blubber thickness (BT)	Physiological	Aug-Dec	May	pan-Baltic	SMNH
Cod	Condition (c)	Physiological	Oct	Oct	BP: SD 25-28<br>BoS; -	ICES,<br>SLU
	Fat content	Physiological	Oct-Nov	Oct-Nov	South eastern Gotland	SMNH
Herring	Weight at age (WAA)	Physiological	all year	all year	BP: SD 25-29, 32<br>BoS: SD 30	ICES
	Fat content	Physiological	BP: Oct<br>BoS: May & October	Oct-Nov	BP: Landsort<br>BoS: Ängskärskubb	SMNH
	Condition (c)	Physiological	Oct-Nov	Oct-Nov	BP; SD 28<br>BoS: SD 30	ICES, SLU
Sprat	Weight at age (WAA)	Physiological	all year	all year	pan-Baltic: SD 22-32	ICES
	Condition (c)	Physiological	Oct-Nov	Oct-Nov	Pan-Baltic: SD 28	ICES, SLU
Saduria entomon	Mean body weight (mw)	Population trait	May	May (Askö)<br>Aug (Outer)	BP: Askö (21 stns), outer (5 stns), BoS: 13 stns	SMHI /<br>SYKE (Table S1)
Monoporeia affinis	Viable embryos per female (ve)	Physiological	Jan	May (see amphipods)	BP: 5 stns<br>BoS: 5 stns	IVL
Amphipods & polychaetes	NA	NA	NA	May	BP: 21 stns<br>BoS: 13 stns	SMHI
Zooplankton	Mean size (ms)	Community trait	June-Sept	June-Sept	BP: Askö and Landsort<br>BoS: 5 stations	SMHI /SYKE (Table S1)

Table S2. Information on sampling stations used for benthos (B) and zooplankton (Z). NBP and WGP denote Northern Baltic Proper and Western Gotland Basin.

Station	Zoopl/Benthos	Country	Assessment unit	Latitude	Longitude	depth	Sampling time	Data originator
SR5	Z/B	Finland	Bothnian Sea	N 61°05.00'	E 19°34.78'	130	Single sampling, August	Finnish Environment Institute
US5	Z/B	Finland	Bothnian Sea	N 62°35.17'	E 19°58.13'	137	Single sampling, August	Finnish Environment Institute
C14	Z	Sweden	Bothnian Sea	N 62°05.29′	E 18°32.95′	85	June-Sep; 3-4 occasions	Umeå University
C3	Z	Sweden	Bothnian Sea	N 62°39.17′	E 18°57.18′	195	June-Sep; 3-4 occasions	Umeå University
F64	Z	Finland	Åland Sea	N 60°11.34'	E 19°08.55'	286	Single sampling, August	Finnish Environment Institute
B1	Z	Sweden	NBP/WGB	N 58°48.19'	E 17° 37' 53	40	June-Sep; 8 occasions	Stockholm University
BY31	Z	Sweden	NBP/WGB	N 58°35.00'	E 18° 14' 00	459	June-Sep; 8 occasions	Stockholm University
LL23	Z	Finland	NBP/WGB	N 58°35.00'	E 18° 14' 00	459	Single sampling, August	Finnish Environment Institute
F69	B	Finland	NBP	N 59°46.95'	E 19°55.95'	193	Single sampling, August	Finnish Environment Institute
LL12	B	Finland	NBP	N 59°28.95'	E 22°.5395	83	Single sampling, August	Finnish Environment Institute
IBSV9	B	Finland	NBP	N 58°25.95'	E 20°.57	88	Single sampling, August	Finnish Environment Institute
IBSV10	B	Finland	NBP	N 58°20.95'	E 21°.151	79	Single sampling, August	Finnish Environment Institute
LF1	B	Finland	NBP	N 57°58.95'	E 21°.1696	68	Single sampling, August	Finnish Environment Institute
6001	B	Sweden	NBP/WGB	N 58° 49.58'	E 17° 34.58	39.5	Single sampling, May	Stockholm University
6004	B	Sweden	NBP/WGB	N 58° 46.44	E 17° 41.48	44	Single sampling, May	Stockholm University

6006	B	Sweden	NBP/WGB	N 58° 43.13	E 17° 50.54	60	Single sampling, May	Stockholm University
6009	B	Sweden	NBP/WGB	N 58° 45.07	E 17° 36.04	17.5	Single sampling, May	Stockholm University
6010	B	Sweden	NBP/WGB	N 58° 50.45	E 17° 33.11	21	Single sampling, May	Stockholm University
6011	B	Sweden	NBP/WGB	N 58° 43.99	E 17° 37.17	27.5	Single sampling, May	Stockholm University
6012	B	Sweden	NBP/WGB	N 58° 47.00	E 17° 34.40	22	Single sampling, May	Stockholm University
6013	B	Sweden	NBP/WGB	N 58° 46.21	E 17° 35.70	9	Single sampling, May	Stockholm University
6014	B	Sweden	NBP/WGB	N 58° 48.17	E 17° 34.71	11	Single sampling, May	Stockholm University
6015	B	Sweden	NBP/WGB	N 58° 46.00	E 17° 39.40	21.5	Single sampling, May	Stockholm University
6016	B	Sweden	NBP/WGB	N 58° 44.68	E 17° 50.20	27	Single sampling, May	Stockholm University
6017	B	Sweden	NBP/WGB	N 58° 46.13	E 17° 45.15	27	Single sampling, May	Stockholm University
6018	B	Sweden	NBP/WGB	N 58° 47.83	E 17° 40.22	21.5	Single sampling, May	Stockholm University
6019	B	Sweden	NBP/WGB	N 58° 44.29	E 17° 41.00	40.5	Single sampling, May	Stockholm University
6020	B	Sweden	NBP/WGB	N 58° 48.66	E 17° 36.41	37	Single sampling, May	Stockholm University
6021	B	Sweden	NBP/WGB	N 58° 46.01	E 17° 49.88	52.5	Single sampling, May	Stockholm University
6022	B	Sweden	NBP/WGB	N 58° 44.64	E 17° 48.84	47	Single sampling, May	Stockholm University
6024	B	Sweden	NBP/WGB	N 58° 47.40	E 17° 45.50	33	Single sampling, May	Stockholm University
6023	B	Sweden	NBP/WGB	N 58° 45.52	E 17° 43.13	36	Single sampling, May	Stockholm University

6025	B	Sweden	NBP/WGB	N 58° 47.42	E 17° 43.70	37.5	Single sampling, May	Stockholm University
N 15	B	Sweden	Bothnian Sea	N 63° 26.25	E 20° 03.00	44.8	Single sampling, May	Umeå University
N 16	B	Sweden	Bothnian Sea	N 63° 24.81	E 19° 51.02	49	Single sampling, May	Umeå University
N 17	B	Sweden	Bothnian Sea	N 63° 23.99	E 19° 58.63	52	Single sampling, May	Umeå University
N 18	B	Sweden	Bothnian Sea	N 63° 22.82	E 19° 44.29	54	Single sampling, May	Umeå University
N 19	B	Sweden	Bothnian Sea	N 63° 21.80	E 19° 44.61	58	Single sampling, May	Umeå University
N 20	B	Sweden	Bothnian Sea	N 63° 22.38	E 20° 00.73	59.4	Single sampling, May	Umeå University
N 21	B	Sweden	Bothnian Sea	N 63° 21.79	E 19° 57.06	61.2	Single sampling, May	Umeå University
N 22	B	Sweden	Bothnian Sea	N 63° 23.35	E 19° 51.44	63.5	Single sampling, May	Umeå University
N 23	B	Sweden	Bothnian Sea	N 63° 20.44	E 19° 40.49	82	Single sampling, May	Umeå University
N 24	B	Sweden	Bothnian Sea	N 63° 25.01	E 20° 06.74	88.5	Single sampling, May	Umeå University
N 25	B	Sweden	Bothnian Sea	N 63° 18.75	E 19° 48.03	126	Single sampling, May	Umeå University

Supplementary Table S3. Detailed results from Kendall-tau tests on status metrics and abundance data from each species/food web component in the respective basin (Bothnian Sea, BoS, or Baltic proper, BP). See figures 3 and 4.

	**BoS**	**BP**
Status metric		
seal BT	K-t: -0.34, p=0.030	
cod c	na	K-t: -0.89, p<0.001
cod fat	na	K-t: -0.49, p=0.002
herring WAA	K-t: -0.34, p=0.030	K-t: 0.12, p=0.446
herring c	K-t: -0.20, p=0.215	K-t: 0.43, p=0.006
herring fat	K-t: 0.07, p=0.693	K-t: 0.33, p=0.032
sprat WAA	na	K-t: -0.01, p=0.933
sprat c	na	K-t: 0.43, p=0.006
Saduria mw	K-t: -0.62, p<0.001	K-t: -0.29, p=0.063
Monoporeia ve	K-t:0.36, p=0.021	K-t: -0.02, p=0.922
zooplankton ms	K-t:- 0.07, p=0.693	K-t: 0.08, p=0.612
Abundance		
seal abund	K-t: 0.97, p=0	
cod abund	na	K-t: 0.20, p=0.215
herring abund	K-t: 0.65, p<0.001	K-t: 0.26, p=0.091
sprat abund	na	K-t: -0.46, p=0.003
Saduria freq (Askö)	na	K-t: 0.37, p=0.024
Saduria freq	K-t: -0.48, p=0.004	K-t: -0.44, p=0.009
Amphipods abund	K-t: -0.43, p=0.006	K-t: 0.26, p=0.102
AmpPol abund	K-t: -0.28, p=0.071	K-t: 0.48, p=0.002
zooplankton biomass	K-t: -0.29, p=0.063	K-t: -0.11, p=0.499

Supplementary Table S4. Predictors in the two best linear models for each response variable (predictors from PLSR results for comparisons, see table 2 and 3 for details). Linear models were tested using a normal error structure and selected based on Akaike information criteria (AIC) estimates and principle of parsimony (within 2 units from lowest AIC). Predictors are listed in order of importance and in bold when there are differences between methods. Analyses were performed in Statistica (Statsoft) using the GLZ module. Goodness of fit and residuals were examined to confirm that the selected error distribution and link function were appropriate.

Response variable	Predictors in PLSR	Predictors in Linear models	AIC
BoS Saduria mw	Amphipods, Freq. Saduria	Amphipods, Freq. Saduria	51.94
		Amphipods	53.39
BP Saduria mw	AmpPol, Freq. Saduria coast	AmpPol, Freq. Saduria coast, Freq. Saduria open sea	58.25
		AmpPol, Freq Saduria open sea	59.89
BP Sprat WAA	Lag Sprat WAA, **zoopl biomass,** cod, **herring,** sprat	Lag sprat WAA, sprat	34.69
		Lag sprat WAA, sprat, cod	36.00
BP Sprat c	Sprat, zooplankton ms, **grey seal**	**Herring**, sprat	33.79
		**Herring,** sprat, zooplankton ms	33.99
BoS Herring fat	Amphipods, zooplankton ms	Zooplankton ms, grey seal, **Herring**	45.96
		Zooplankton ms, Amphipods	46.12
BoS Herring WAA	Amphipods, **zooplankton ms,** grey seal	Amphipods	53.52
		Amphipods, **herring**, grey seal	54.11
BoS herring c	Amphipods, **zooplankton ms**	Amphipods, grey seal	51.58
		Amphipods, **herring**	52.23
BP herring fat	Sprat, grey seal, zooplankton **ms**	**Freq. Saduria**, grey seal, sprat, **herring**, zooplankton **biomass**	51.08
		Sprat	52.10
BP Herring WAA	Lag herring WAA, sprat	Lag herring WAA, **AmpPol**, sprat, **herring**	27.17
		Lag herring WAA, zooplankton biomass, AmpPol, sprat, herring	28.98
BP herring c	Sprat, grey seal	Grey seal, **cod**, sprat, **herring**	36.37
		Sprat, grey seal, **herring**	37.31
Cod fat	Cod, **grey seal**	**Herring WAA, sprat, sprat WAA** cod, **Freq Saduria open**	51.58
		**Herring WAA, sprat WAA**, cod, **Freq Saduria open**	52.10
Cod c	Grey seal, **Freq. Saduria open**	**Herring, Herring WAA,** grey seal	26.03
		**Herring,** grey seal	26.73
Grey seal BT	BOS Herring WAA, cod **c**, grey seal	BOS herring **c,** BOS herring WAA, BOS herring **fat, sprat WAA,** cod **fat, sprat, cod,** grey seal, **herring BOS**	46.12
		BOS herring **c,** BOS herring WAA, BOS herring **fat, sprat WAA**, cod **fat**, **sprat, cod**, grey seal	47.41

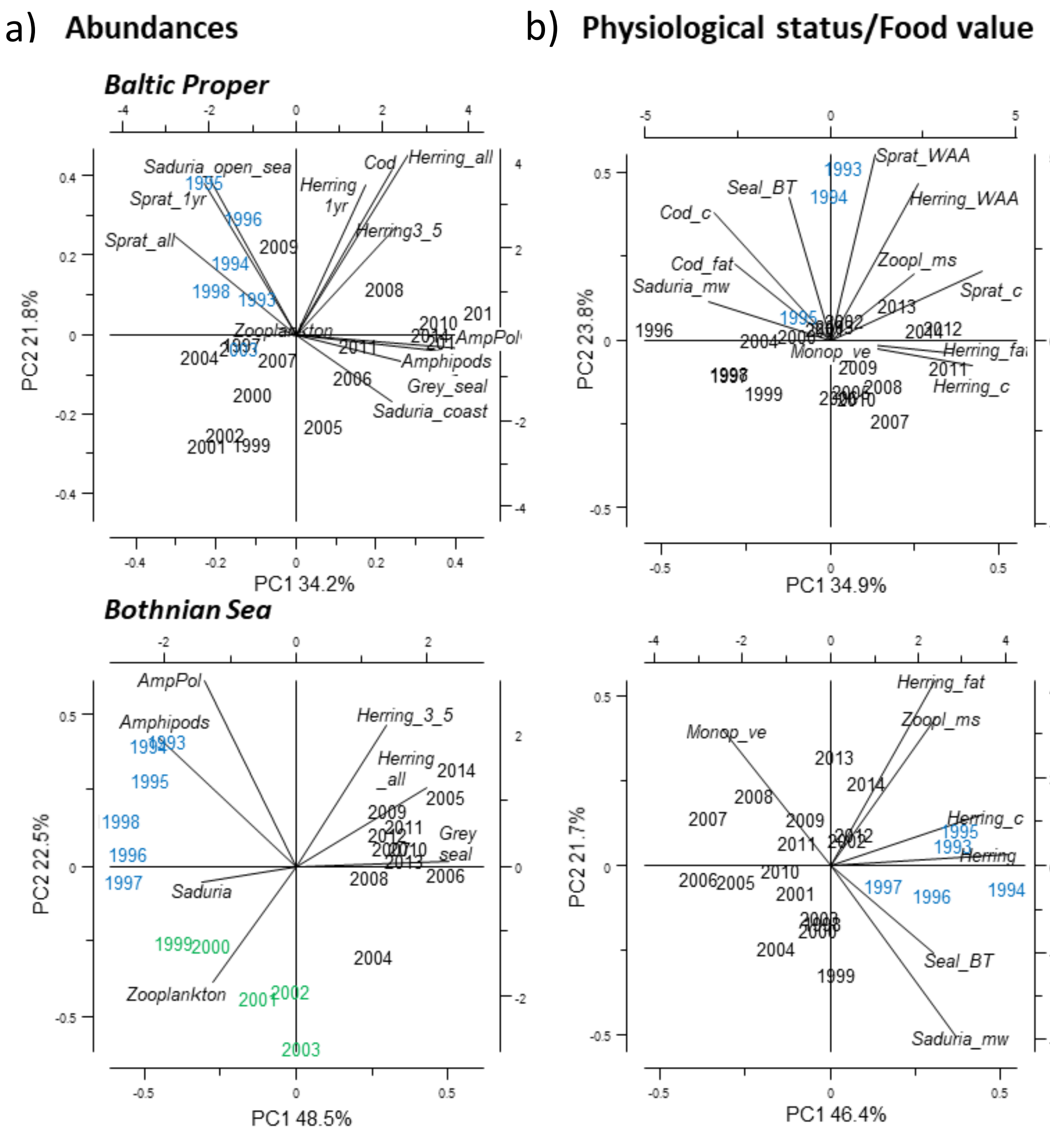

Figure S1. PCA biplots showing sample (year) scores and vectors for the assessed metrics. Abundance metrics (a) are shown at the left side of the panel and metrics representing physiological status or population/community traits ("food value") (b) on the right side, separately for the Baltic proper (upper part of panel) and Bothnian Sea (lower part). Similarities and sudden shifts between adjacent years were assessed by chronological clustering. Similar colors for the years indicate higher similarity, while years with different colours are separated ($p<0.01$) based on euclidean distances. In both sub-basins, these analyses show a shift between the earlier years studied (until years between 1996 and 1998 for the different plots) for both physiological status/food value and abundances, reflecting changes in the relative abundance of taxa from different trophic levels and a decreasing physiological status/food value. Physiological status metrics are abbreviated as c (condition, see equation 1), WAA (weight at age) and ve (viable embryos, *Monoporeia affinis*). Population and community traits, respectively, are mw (mean weight, *Saduria entomon*), ms (mean size, zooplankton). For abundance data of herring and sprat, data are shown for their respective different year classes used in analyses (compare Table 3, e.g. 1 and 3-5 year olds, as well as the summed abundance of all year classes).
